# Local Tiled Deep Networks for Recognition of Vehicle Make and Model

**DOI:** 10.3390/s16020226

**Published:** 2016-02-11

**Authors:** Yongbin Gao, Hyo Jong Lee

**Affiliations:** 1 Division of Computer Science and Engineering, Chonbuk National University, 567 Baekje-Daero, Deokjin-Gu, Jeonju 54596, Korea; gaoyongbin.sam@gmail.com; 2 Center for Advanced Image and Information Technology, Chonbuk National University, 567 Baekje-Daero, Deokjin-Gu, Jeonju 54596, Korea

**Keywords:** moving-vehicle detection, vehicle-model recognition, deep learning, HOG

## Abstract

Vehicle analysis involves license-plate recognition (LPR), vehicle-type classification (VTC), and vehicle make and model recognition (MMR). Among these tasks, MMR plays an important complementary role in respect to LPR. In this paper, we propose a novel framework for MMR using local tiled deep networks. The frontal views of vehicle images are first extracted and fed into the local tiled deep networks for training and testing. A local tiled convolutional neural network (LTCNN) is proposed to alter the weight sharing scheme of CNN with local tiled structure. The LTCNN unties the weights of adjacent units and then ties the units *k* steps from each other within a local map. This architecture provides the translational, rotational, and scale invariance as well as locality. In addition, to further deal with the colour and illumination variation, we applied the histogram oriented gradient (HOG) to the frontal view of images prior to the LTCNN. The experimental results show that our LTCNN framework achieved a 98% accuracy rate in terms of vehicle MMR.

## 1. Introduction

Vehicle analysis is widely used in various applications such as driver assistance, intelligent parking, self-guided vehicle systems, and traffic monitoring (quantity, speed, and flow of vehicles) [1]. A notable example is an electronic tollgate system that can automatically collect tolls based on the identification of vehicle’s make and model. This MMR technology can be further used for public security to monitor suspicious vehicles.

The detection of moving vehicles is the first task of a vehicle analysis. Background subtraction [2,3,4,5] plays an important role by extracting motion features from video streams for detection; however, this algorithm cannot be applied to static images. Many studies tried to solve this problem including a Wu *et al.* study in which a wavelet was used to extract texture features to localize candidate vehicles [6]. Furthermore, Tzomakas and Seelen suggested that the shadows of vehicles are an effective clue for vehicle detection [7]. Ratan *et al.* used the wheels of vehicles to detect candidate vehicles and then refined the vehicles using a diverse density method [8].

The tasks of vehicle analysis include license plate recognition (LPR), vehicle-type classification [9], and vehicle MMR [10]. LPR provides a unique identification for a detected vehicle, but the technique is not reliable due to the LPR-specific errors that can be incurred by low resolution, bad illumination, or fake license plates. Vehicle-type classification attempts to distinguish vehicles according to identifiers such as “saloon”, “estate vehicle”, and “van”, while vehicle MMR tries to identify the manufacturer and model of a vehicle, such as “Kia K3” or “Audi A4”. Vehicle-type classification and vehicle MMR therefore provide valuable complementary information in the case of an LPR failure. They also contribute to public security through the detection of suspicious vehicles and the identification of improper driving.

However, the distinction of vehicle with same manufacturer, model, and reflective coating is an unresolved and challenging problem in MMR technique. Abdel Maseeh *et al.* incorporated global and local cues to recognize vehicle models [11], while Hsieh *et al.* proposed a symmetrical Speeded Up Robust Features (SURF) method for both vehicle detection and MMR [12]. The frontal or rear view image is widely used for the Region of Interest (ROI) of MMR due to its computational efficiency and discriminative property; Petrovic and Cootes first proposed this concept for MMR [13], which established the baseline and methodology for later research studies [14,15]. Llorca proposed the use of geometry and the rear-view image of a vehicle for vehicle-model recognition [16]. Considering the 3D properties of a vehicle, 3D-based methods [17,18] have been proposed to enable vehicle MMR from an arbitrary view, including a 3D view-based alignment method to match 3D curves to 2D images [18]. A significant issue with 3D object recognition is large variance within one model that is incurred by view changes. Other research studies using various features and machine-learning techniques such as SIFT [19], Harris corners [20], and texture descriptors [21,22,23] were also developed. In terms of a classifier, SVM [14], nearest neighbour [15], and neural networks [24] were widely used. 

In this paper, we propose a framework for vehicle MMR based on local tiled CNN. Deep learning based on CNN has achieved state-of-the-art performance in various applications, including handwritten digit recognition [25] and facial recognition [26]. CNN uses the hard-coded weight sharing scheme with translational invariance that prevents network from capturing more complex invariance. The proposed LTCNN unties the weights of adjacent units and ties units that are k steps away from each other within a local map. This alternate architecture provides the translational, rotation, and scale invariance as well as locality. In addition, to further deal with the colour and illumination variation, we applied the histogram of oriented gradient (HOG) to the frontal view of images prior to the LTCNN. 

The remainder of this paper is organized as follows: Section 2 introduces the related work in terms of convolutional neural network; Section 3 describes the framework of our MMR system in terms of moving-vehicle detection and a frontal-view extraction method; we then introduce the vehicle-model recognition based on LTCNN in Section 4; Section 5 describes the histogram of orientated gradient algorithm; Section 6 applies the previously mentioned algorithm to our vehicle database and presents the experiment results; and lastly, Section 7 presents the conclusions of our paper.

## 2. Related Work

Deep networks are used to extract discriminative features for vehicle MMR. The deep network concept has been around since 1980, with similar ideas including neural network and back propagation. The resurgence of interest on deep networks is brought on by breakthrough in Restricted Boltzmann Machines (RBM) from Hinton [27]. For the last three years deep learning-based algorithms have won the ImageNet Large Scale Visual Recognition Challenge (ILSVRC), which used convolutional architectures of deep models.

### 2.1. RBM and Auto-Encoder

RBM and auto-encoder are two primary division for deep learning, the former uses the probabilistic graphical models while the latter roots in computation graphs. A deep network is more capable of characterizing input data with multiple layers. Training a deep network with traditional back-propagation suffers from problems of poor local optima and high time-consumption. Another problem is the requirement of labeled data for back-propagation even though labeled data are not always available for small datasets. Alternatively, the deep-learning method [27] uses unlabeled data to initialize the deep model, thereby resolving the poor local optima and lengthy time frame through learning of the p(image) instead of the p(label|image). This technique generates input data through maximizing probability of generative model.

Unlabeled data are easier to acquire than labeled data. We therefore used unlabeled data for the pre-training of the deep architecture to obtain the initial weights. Through these initial weights that are in the region of an effective solution, an optimal solution is readily accessible. An RBM provides us with an effective pre-training method that is comprised of a two-layer network with stochastic, binary pixels as units; these two layers consist of pixels of “visible” units and “hidden” units that are connected with symmetrically weighted connections. 

The RBM has been extended to Gaussian RBM (GRBM) to enable the real-valued data by modelling visible units as a Gaussian distribution [28]. The mean and covariance RBM (mcRBM) was proposed to parametrize the mean and covariance from hidden units [29]. For natural images, mPoT model [30] was used to extract large-scale features. As for the auto-encoder, sparse auto-encoders are proposed to regularize the sparsity, such as Contractive auto-encoders (CAE) [31] and Denoising auto-encoders (DAE) [32].

### 2.2. Convolutional Models

Massive parameters are involved in the deep networks, which requires a large scale dataset to training. To this end, convolutional structure is usually adopted to reduce the number of parameters, such as convolutional neural network (CNN) [33] and convolution RBM [34]. In addition, many advanced techniques have been coupled into the CNN structure, such as dropout, maxout, max-pooling. By going deeper with the convolutional networks, CNN dominates the performance in various applications, such as AlexNet [35], Overfeat [36], GoogLeNet [37], and ResNet [38].

**Figure 1:**
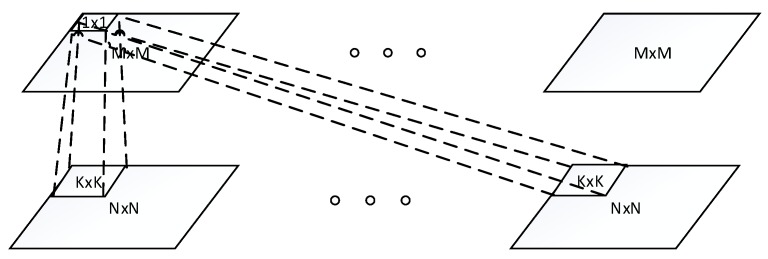
Architecture of one convolutional layer.

The architecture of one convolutional layer is illustrated in the Figure 1, where the bottom layer is N×N input layer, and the top layer is M×M convolutional layer. The convolutional layer conducts convolution operation across the input maps with a K×K filter for each map, resulting in a (N−K+1)×(N−K+1) feature map. Thus, M=N−K+1. 

**Figure 2:**
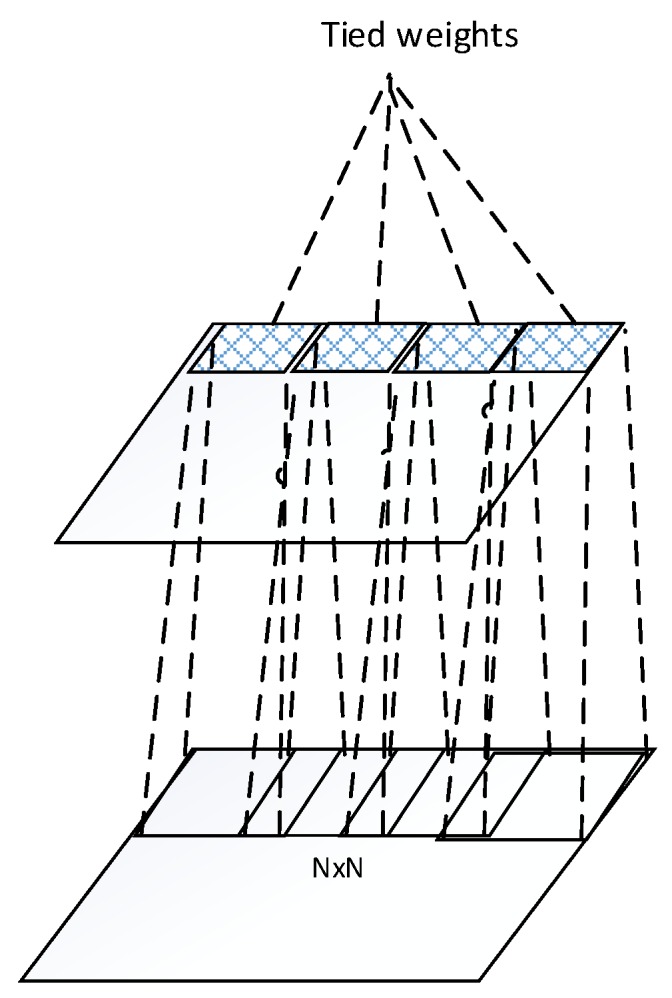
Tied weights within a feature map of a convolutional layer.

The idea behind the convolution layer is weight sharing within a feature map as shown in Figure 2. The units of a feature map are weights tied. Through this scheme, the number of weights to be trained is reduced significantly. This hard-coded network provides translational invariance by pooling across units of each feature map. However, more invariances towards rotation and scale change are disabled by this hard-coded structure. 

## 3. Framework of Proposed MMR System

**Figure 3:**
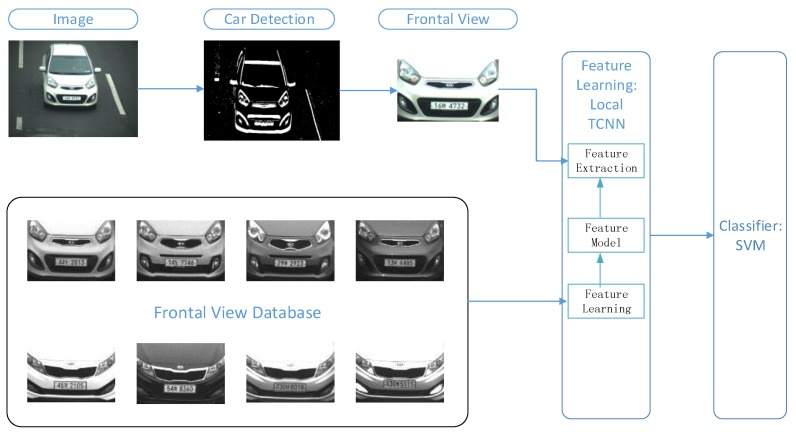
Framework of proposed vehicle make and model recognition (MMR) system based on LTCNN. Moving vehicle was detected using the frame difference, and the resultant binary image was used to detect the frontal view of a vehicle with a symmetry filter. The detected frontal view was used to identify the vehicle make based on the LTCNN algorithm.

A correct frontal view is essential for the deep-learning training. Figure 3 shows the proposed MMR system based on LTCNN. Each vehicle model consists of multiple samples for the training of the deep network; based on the trained model, we were able to recognize the vehicle make and model. The proposed MMR system involves the following three major steps: (1) Vehicle detection from a video or image; (2) ROI extraction, which is the frontal view of a vehicle in our system; and (3) vehicle MMR.

Vehicle detection is the first step of an MMR system. In this study, we used frame difference to detect a moving vehicle because our camera is static on the streets. Frame difference is efficient in terms of the computational power that enables real-time application in this scenario. 

We applied only the frontal view of a vehicle for real-time application in MMR processing, while 3D MMR [18] used an entire vehicle image. The frontal view images of vehicle provide sufficient discrimination to recognize vehicle models, as shown in many other studies [14,15]. Meanwhile, the frontal view is relatively small compared to the entire vehicle image, which reduces the computational time. Notably, the frontal view of a vehicle is typically symmetrical, thus a symmetrical filter was used to detect the frontal view in our system. The symmetrical filter first sums up the values of pixels of the same column. The car image turns to be a vector, and the symmetrical filter is performed in a sliding-window manner by calculating the difference between left and right parts of the window. The window of the minimal difference is regarded as the frontal view of a car. 

The LTCNN was performed on the HOG features of the frontal view of the vehicles for vehicle MMR. HOG extracts the texture of images that is consequently more robust against geometric and illumination variations. LTCNN learns the feature model from the frontal view database, the features of new image are extracted based on the feature model. The extracted features are further fed into the large linear SVM [39].

## 4. Local Tiled Convolutional Neural Networks

**Figure 4:**
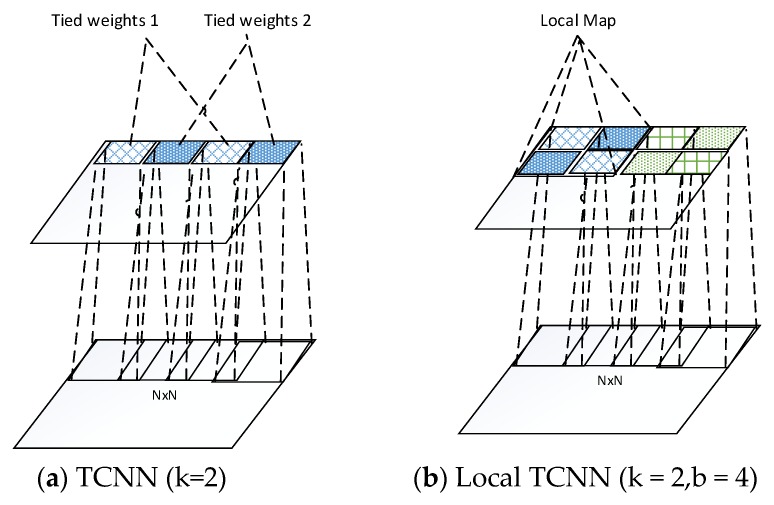
The convolutional layer of TCNN and proposed local TCNN. Units with same colour and fill are weights tied.

As we pointed out in the related work section, CNN only provides the translational invariance. However, in real world, the input images varies with rotation and scale. The CNN is not capable of capturing these variances since the pooling layer only pool over the same basis/filter. To address this problem, a tiled CNN (TCNN) [40] is proposed by using only tied units that are *k* steps away from each other as shown in Figure 4a, where *k* = 2 shows that the stripe between two tied units is 2. This enables the adjacent untied units, which refers to as “tiling.” By varying the stripe *k*, TCNN learns various structure of models that provides rotation and scale invariances. The TCNN inspired us to consider the locality of images. As we know, the convolutional operation between layers is essentially a linear function. The conventional CNN/deep learning ties the weights for the entire upper layer, which characterizes only one linear transformation for the whole input. However, the local map with different characteristics and features tends to have varying transformation goals through the network, which can handle more variations of the input data. By untying the weights between different local maps, LTCNN is capable of learning various linear function for different local maps. It is meaningful to warp the different local map with different weights especially in case the input data vary in pose or angle. In this paper, we extended the idea of TCNN and coupled with locality of images. Local part of an image tends to share the weights, while units that far apart from each other do not have sharing weights. Following this idea, we develop a novel algorithm, which we call “local tiled CNN” (LTCNN) as shown in Figure 4b. The feature map of the convolutional layer is divided into identical local maps, within each local map is a tiny TCNN. For example, when tied stripe *k* = 2, and the number of local map is *b* = 4, there are totally eight tiled units for each feature map, which learns eight basis/filters for each feature. This various basis provide not only the translational, scale, rotation invariance, but also the locality of feature maps. Specially, when *k* = 1, and *b* = 1, LTCNN decays to the typical convolutional layer. On the other hand, when $k\times b=M^{2}$, where $M^{2}$ is the number of units in the feature map, LTCNN turns out to be traditional neural network of untied units.

**Figure 5:**
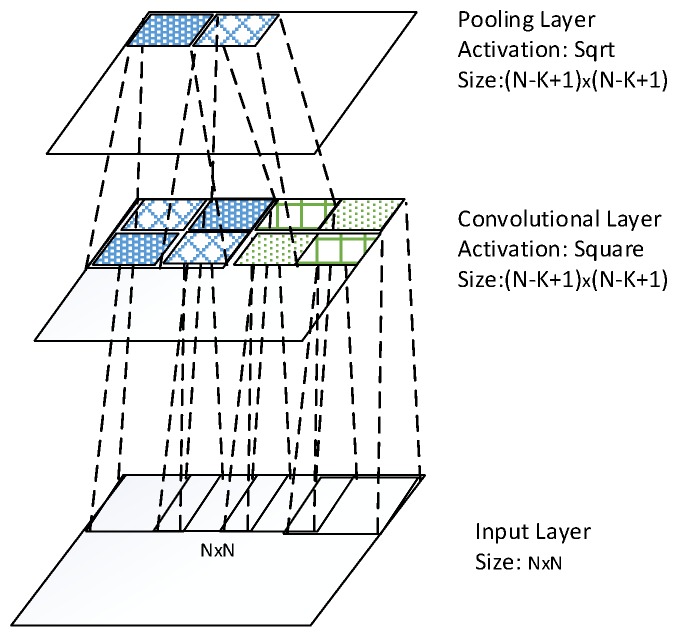
Architecture of TICA network.

Unsupervised feature learning is used to extract features in this study. In order to perform LTCNN in an unsupervised manner, we use Topographic Independent Component Analysis (TICA) network [41] as in the study [40]. Our LTCNN is integrated into the TICA network, which has three layers network: input layer, convolutional layer, and pooling layer as shown in Figure 5, where the convolutional layer is altered to our LTCNN layer, and the pooling layer is connected to a small patch of the convolutional map to pool over the adjacent units. For convenience, we unroll the 2D map into vector as a representation. The weights W between input pattern $x^{t}$ and convolutional layer ${\left\{h_{i}\right\}}_{i=1}^{m}$ are to be learned, while the weights V between convolutional layer and pooling layer ${\left\{p_{i}\right\}}_{i=1}^{m}$ are fixed and hard-coded, where $W\in R^{m\times n}$ and $V\in R^{m\times m}$, m and n is the number of convolutional units and input size. The output units of the second layer can be described as:

(1) $$p_{i}\left(x^{t},W,V\right)=\sqrt{\overset{m}{\underset{k=1}{\sum }}V_{ik}{\left(\overset{n}{\underset{j=1}{\sum }}W_{kj}x_{j}^{t}\right)}^{2}}$$

where $V_{ik}=1\text{ or }0$ encodes whether the pooling units i is connected to convolutional units k or not, and if the convolutional unit is not connected to input units, $W_{kj}=0$. The goal of the TICA is to find the sparse representations of the output features, which can be solved by:

(2) $$\underset{W}{\text{min}}\overset{T}{\underset{t=1}{\sum }}\overset{m}{\underset{i=1}{\sum }}p_{i}\left(x^{t},W,V\right),\text{ subject to }WW^{T}=I$$

where the condition of $WW^{T}=I$ ensures that the learned features are competitive and diverse. As the weights between convolutional layers connect with local receptive field, it is constrained to zero outside the small receptive field. The weights of different receptive fields are orthogonal. Thus, the remaining problem is to ensure the weights that are on the same local receptive field to be in similar orthogonal orientation. This is more efficient than the primitive TICA. The weights W can be learned by stochastic gradient descent and back propagation, the only difference is the weights orthogonalization after each update of weights.

**Figure 6:**
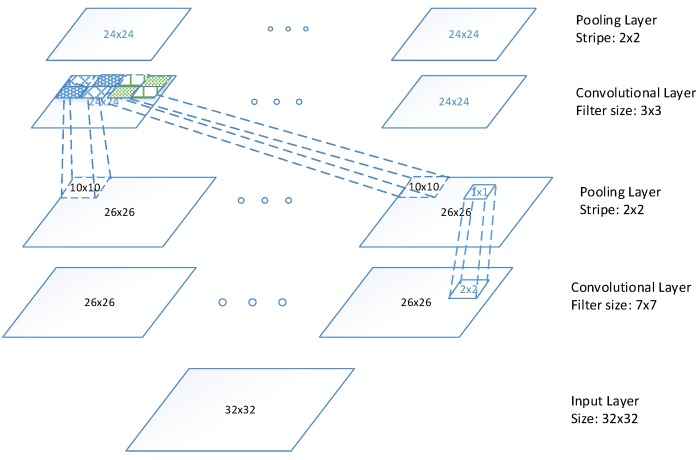
Architecture of network based on the proposed LTCNN.

The overall architecture of our network is illustrated in Figure 6. The input frontal views of car images are resized to 32×32, and feed into two TICA network, resulting in a four layer network. The first and third layers are LTCNN with filter size 7×7, and 3×3, respectively. The second and forth layers are pooling layer with stripe of 2×2. The resulting feature map is 24×24.

## 5. Histogram of Oriented Gradient

To further improve the invariance of LTCNN algorithms towards the colour and illumination, we apply the histogram of oriented gradient (HOG) to the frontal views of images. The idea behind HOG features is the characterization of the appearance and shape of the local object using the distribution of local intensity gradients or edge directions. The local property is implemented by the division of the image into cells and blocks. The main HOG steps are summarized as follows:

(1) Gamma/Color Normalization: Normalization is necessary to reduce the effects of illumination and shadow; therefore, gamma/colour normalization is performed prior to the HOG extraction.
(2) Gradient Computation: Gradient and orientation are calculated for further processing, as they provide information regarding contour and texture that can reduce the effect of illumination. Gaussian smoothing followed by a discrete derivative mask are effective for calculating the gradient. For the color image, gradients are calculated separately for each color channel, and the one with the largest norm is considered the gradient vector.
(3) HOG of cells: Images are divided into local spatial patches called “cells”. An edge orientation histogram is calculated for each cell based on the weighted votes of the gradient and orientation. The votes are usually weighted by either the magnitude of gradient (MOG), the square of MOG, the square root of MOG, or the clipped form of MOG; in our experiment, the function of MOG was used. Moreover, the votes were linearly interpolated into both the neighbouring orientation and position.
(4) Contrast normalization of blocks: Illumination varies from cell to cell resulting in uneven gradient strengths, and local contrast normalization is essential in this case. By grouping cells into a larger spatial block with an overlapped scheme, we were able to perform contrast normalization at a block level.

## 6. Results

To evaluate the performance of the proposed algorithm, we built a vehicle make and model dataset. This dataset comprises 3210 vehicle images with 107 vehicle models, and 30 images of various colors and illuminations are captured for each model. The specifications of the computer used to perform all of the experiments are as follows: Intel Core i7-4790 with 3.6 GHz and 8 GB of RAM, running on 64-bit Windows 7 Enterprise SP1.

### 6.1. Frontal-View Extraction

A frame-difference algorithm was used for the moving-vehicle detection in our study, while the database consists of images that makes it easier than video to measure the accuracy. To make frame difference works for the images, we generated an adjacent frame by shifting each of the images by 10 pixels. The frame difference is performed between the original image and its shifted image in our system. The frame difference is integrated with a mathematical morphology operation and the symmetrical filter to find the exact location of frontal view of a car.

**Figure 7:**
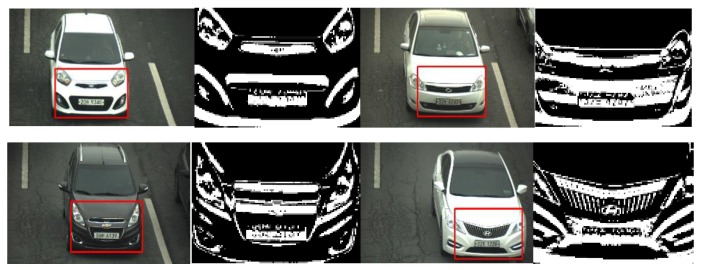
Results of frontal-view extraction on five vehicle images featuring four companies and four models.

Figure 7 presents the results of the frontal-view extraction including four vehicle models of four companies. The red rectangle in the original vehicle image is the detected frontal view of the vehicle, and the binary image of each frontal view is also presented. The experimental results illustrate that our system is capable of accurately extracting the frontal view of vehicles. The algorithm was evaluated over 3210 images, and achieved 100% accuracy in terms of frontal-view extraction.

### 6.2. Local Tiled CNN

**Figure 8:**
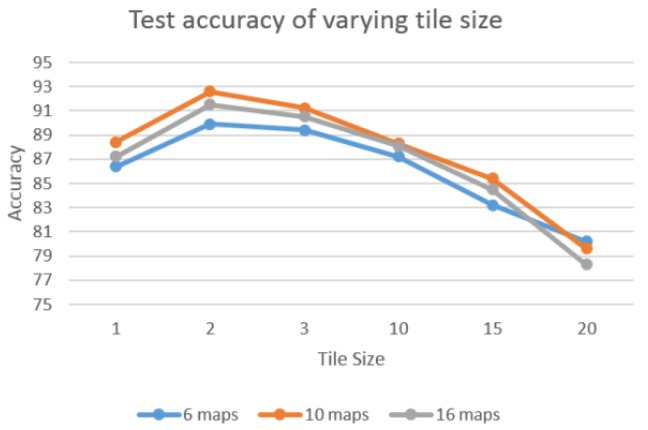
Test accuracy of varying tile size with fixed number of local maps (16).

**Figure 9:**
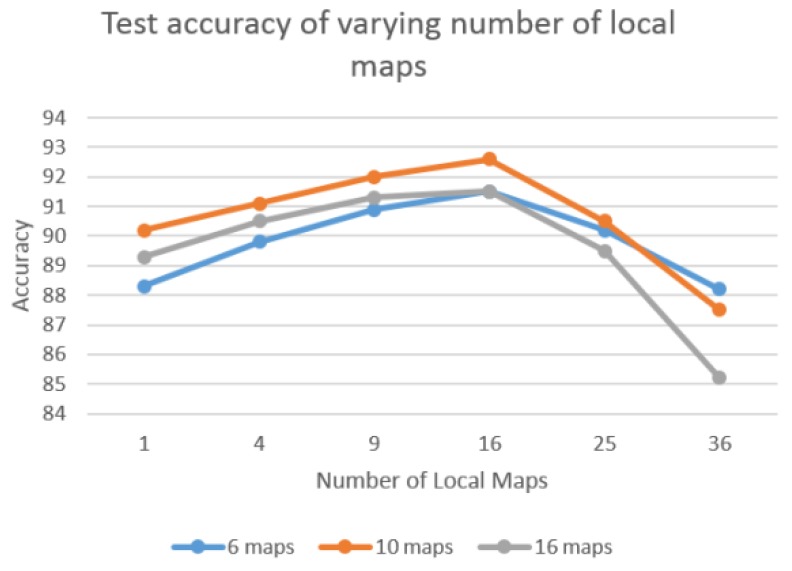
Test accuracy of varying number of local maps with fixed tile size (2).

Local tiled CNN involves numerous crucial parameters, such as the number of maps, local maps, and tile size. To investigate the effect of different parameters, we evaluate the performance of varying parameters. For each experiment, we vary one parameter and fix the others. Figure 8 shows the accuracy of varying tile size when the number of local maps is fixed as 16. The various tile sizes are tested as {1, 2, 3, 10, 15, 20}. Result indicates that tile_size = 2 achieved the best performance among these experiments. This is because the increasing tile size tends to overfit since the number of training samples are limited. Figure 9 illustrates the test accuracy of varying number of local maps with fixed tiled size as 2. The test series of various number of local maps are {1,4,9,16,25,36}. The results show the number of local maps set to 16 achieve the best performance. This shows the same pattern as Figure 8, in which the bigger value does not necessarily result in better performance. Similarly, 10 maps show the best performance for both Figure 8 and Figure 9. Thus, in our experiments, we set number of maps, number of local maps, and tiled size as 10, 16, and 2, respectively.

### 6.3. Enhancement with HOG Feature

**Figure 10:**
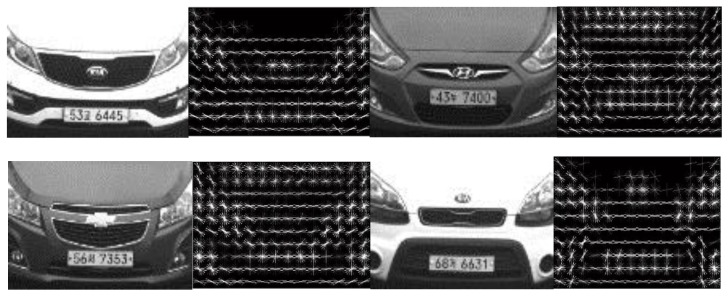
HOG features of frontal view of vehicle image, cell size was set to 8 × 8, and the size of the HOG features is 10 × 14 × 36.

**Figure 11:**
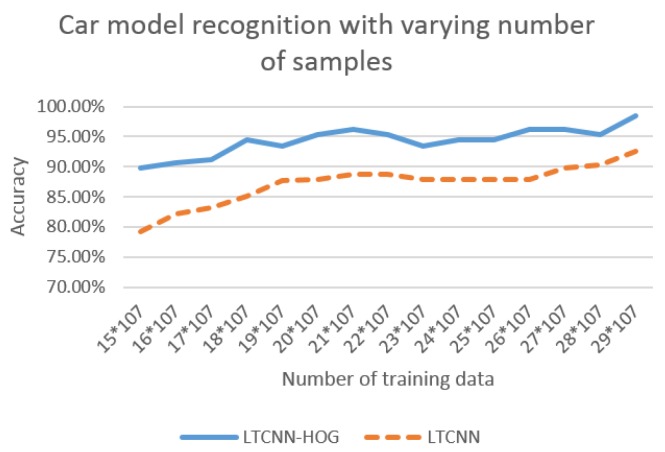
Accuracy of the vehicle-model recognition with different number of sample sizes for each experiment. The size of cell and HOG features are set to 10 × 14 × 36, respectively.

HOG features were extracted from the detected frontal views. Figure 10 shows the HOG features of the frontal views of the vehicle images. The frontal image was resized to 80 × 112 and HOG was applied to the resized image with 8 × 8 cell size in our experiment. The size of the resultant HOG features is therefore 10 × 14 × 36. This allows 140 cells with a descriptor of dimension by 36 for each cell. Figure 11 illustrates that the HOG features characterized the orientations of the frontal view well, and frontal images that are discriminative with each other are more effective.

To evaluate the performance of our LTCNN with HOG, we first compared LTCNN-HOG with LTCNN using varying numbers of training samples; we secured 30 samples for each vehicle model for the purpose of reasonable training. We divided the samples into a training set and a testing set, varying the number of training samples between 15 and 29. For each experiment, the accuracy of the vehicle-model recognition was calculated and plotted, as shown in Figure 11; this figure shows that LTCNN-HOG outperformed LTCNN regardless of the number of training samples. It is noted that both curves show an increased accuracy that corresponds with an increased sample number. There is approximately a 5% accuracy difference between 15 training images and 29 training images for both algorithms.

### 6.4. Comparison with Other Algorithms

Lastly, we compared our LTCNN method to the following widely used algorithms: local binary pattern (LBP) [22], local Gabor binary pattern (LGBP) [23], scale-invariant feature transform (SIFT) [19], linear SVM [39], RBM [27], CNN [35], TCNN [40]. Specifically, the experiment protocol of feature extraction based methods are interpreted in detail, the other algorithms have the same experimental protocol with LTCNN.

(1) LBP: The LBP operator, one of the most popular texture descriptors of various applications, is invariant to monotonic changes and computational efficiency. In our experiment, frontal-view images were characterized by a histogram vector of 59 bins, and the weighted Chi-square distance was used to measure the difference between the two LBP histograms.
(2) LGBP: LGBP is the extension of LBP incorporated with a Gabor filter, whereby the vehicle images are first filtered using Gabor and the results are in Gabor Magnitude Pictures (GMPs) frequency domain. In our experiment, five scales and eight orientations were used for the Gabor filter; as a result, 40 GMPs were generated for each vehicle image. Also, the weighted Chi-square distance was used to measure the differences between the LGBP features.
(3) SIFT: SIFT is an effective local descriptor with scale and rotation-invariant properties. Training is not necessary for the SIFT algorithm. To compare SIFT with our LTCNN that requires training in advance, we used the same number of training sets to make a comparison with a test image, and a summation of the matched keypoints was used to measure the similarities between the two images.

In our experiments, 29 images of each vehicle make were used for training, and the remaining images were used for testing. The comparison results are shown in Table 1. The results show that LTCNN with HOG achieved the best performance among the compared methods. Our LTCNN outperformed the feature extraction based methods significantly. LTCNN also achieved an accuracy that is 5% higher than CNN/RBM. It is also noted that the 5% accuracy rate was gained by incorporating HOG features into the LTCNN method. The computational time is also shown in Table 1. The LTCNN increases the accuracy without significantly increased computional time. The neural network based algorithms are more scalable towards the increasing number of the classes. In contrast, LBP, LGBP, SIFT suffers from the long computational time when it comes to large size of images.

**Table 1:** Performance comparison of vehicle-model recognition with prestigious methods.

Algorithm	Accuracy (%)	Time (ms)
LBP	46.0	2385
LGBP	68.8	3210
SIFT	78.3	3842
Linear SVM	88.0	1875
RBM	88.2	539
CNN	88.4	1274
TCNN	90.2	843
LTCNN	93.5	921
LTCNN (with HOG)	98.5	1022

## 7. Conclusions

In this paper, a framework for vehicle MMR that is based on LTCNN is proposed. We first detected moving vehicles using frame difference; the resultant binary image was used to detect the frontal view of a vehicle using a symmetry filter. The detected frontal view was used to identify a vehicle based on LTCNN. The frontal view of the vehicle images were first extracted and characterized using the HOG features; the HOG features were fed into the deep network for training and testing. The results show that LTCNN with HOG achieved the best performance among other comparable methods. Our LTCNN outperforms the feature extraction based methods significantly. The accuracy of proposed MMR algorithm is 5% higher than the CNN or RBM. Another 5% of accuracy is gained by incorporating HOG features into the LTCNN method. Thus, the accuracy of LTCNN with HOG features shows 10% higher than traditional approaches. Furthermore, computational time of proposed method takes only 66% of CNN, which means that real-time recognition is possible. 
